# Adhesive chitosan-based hybrid biohydrogels for peripheral nerve injury repair

**DOI:** 10.3389/fcell.2024.1499766

**Published:** 2024-11-14

**Authors:** Pengjia Qiu, Lei Wang, Jing Wang, Xingdong Wang, Jianchao Xu, Xiaokai An, Fengwang Han, Zhao Dong, Jiangtao Zhang, Peiwen Shi, Qiang Niu

**Affiliations:** ^1^ Department of Orthopedics, Gaoyang County Hospital, Baoding, Hebei Province, China; ^2^ Department of Orthopedics, Sichuan Gemflower Hospital, North Sichuan Medical College, Sichuan, China

**Keywords:** traumatic injury, adhesion materials, chitosan-based hydrogel, hybrid biohydrogel, peripheral nerve injury

## Abstract

With the rapid progress of industrialization, the incidence of peripheral nerve injuries caused by trauma has been continuously increasing. These injuries result in a significant number of disabilities and irreversible functional impairments, not only severely impacting the health and quality of life of patients but also placing a heavy economic burden on families and society. Effectively promoting peripheral nerve regeneration has thus become a key focus and challenge in current research. In recent years, hybrid biohydrogels with adhesive properties have gained widespread attention due to their excellent biocompatibility, mechanical stability, conductivity, and biodegradability. These materials can provide an optimal microenvironment to promote neuron adhesion and axonal extension while offering outstanding mechanical strength to meet the fixation requirements in clinical surgeries. This paper systematically reviews the application of adhesive hybrid biohydrogels in peripheral nerve injury repair, highlighting the latest research progress in promoting nerve regeneration and improving functional recovery, and discusses the challenges and future prospects for their clinical application.

## 1 Introduction

With the increasing number of traffic accidents, medical accidents, and the rapid expansion of mechanized production, traumatic peripheral nerve injury occurs more frequently (Hewson et al., 2018; Huckhagel et al., 2018; Laumonerie et al., 2018). Once it happens, it may lead to sensory and motor dysfunction, seriously affect the body’s function and daily life, and cause significant burden and pressure on social and personal life. The structure and function of human nerve tissue are complex and changeable, so it is difficult to repair completely (Li et al., 2022; Liu et al, 2022). Clinically, the first choice for treating peripheral nerve injury is a tension-free epineural suture after docking short-distance injured nerves under microsurgery (Pauchot et al., 2017). For long-distance peripheral nerve injuries, autologous and allogeneic nerve grafts are generally used (Bellaire et al., 2021; Caronna et al., 2021; DeLeonibus et al., 2021; Kornfeld et al., 2021; Wang et al., 2021). The disadvantages of autologous nerve transplantation are limited nerve source, easy damage or permanent loss of donor function, limited repairable length, long operation time, the low success rate of transplantation, the emergence of the painful neuroma, and so on (Gao et al., 2015; Wilson, 2021). Autogenous nerve transplantation still does not achieve satisfactory results in clinical repair and will still produce serious complications (Bastami et al., 2017; Sebben et al., 2011). Similarly, allogeneic nerve transplantation is more likely to appear immune and rejected, which is not conducive to recovery from peripheral nerve sensation and function (Huang et al., 2019; Kornfeld et al., 2019). Therefore, many researchers use hybrid biohydrogels to repair peripheral nerves (Dolkhani et al., 2020; Ma et al., 2021; Uranues et al., 2020). They were hoping to develop a perfect material to repair peripheral nerve injury to alleviate the pain of patients. Through the efforts of many experts and scholars, a variety of nerve substitute materials have been found.

**FIGURE 1:**
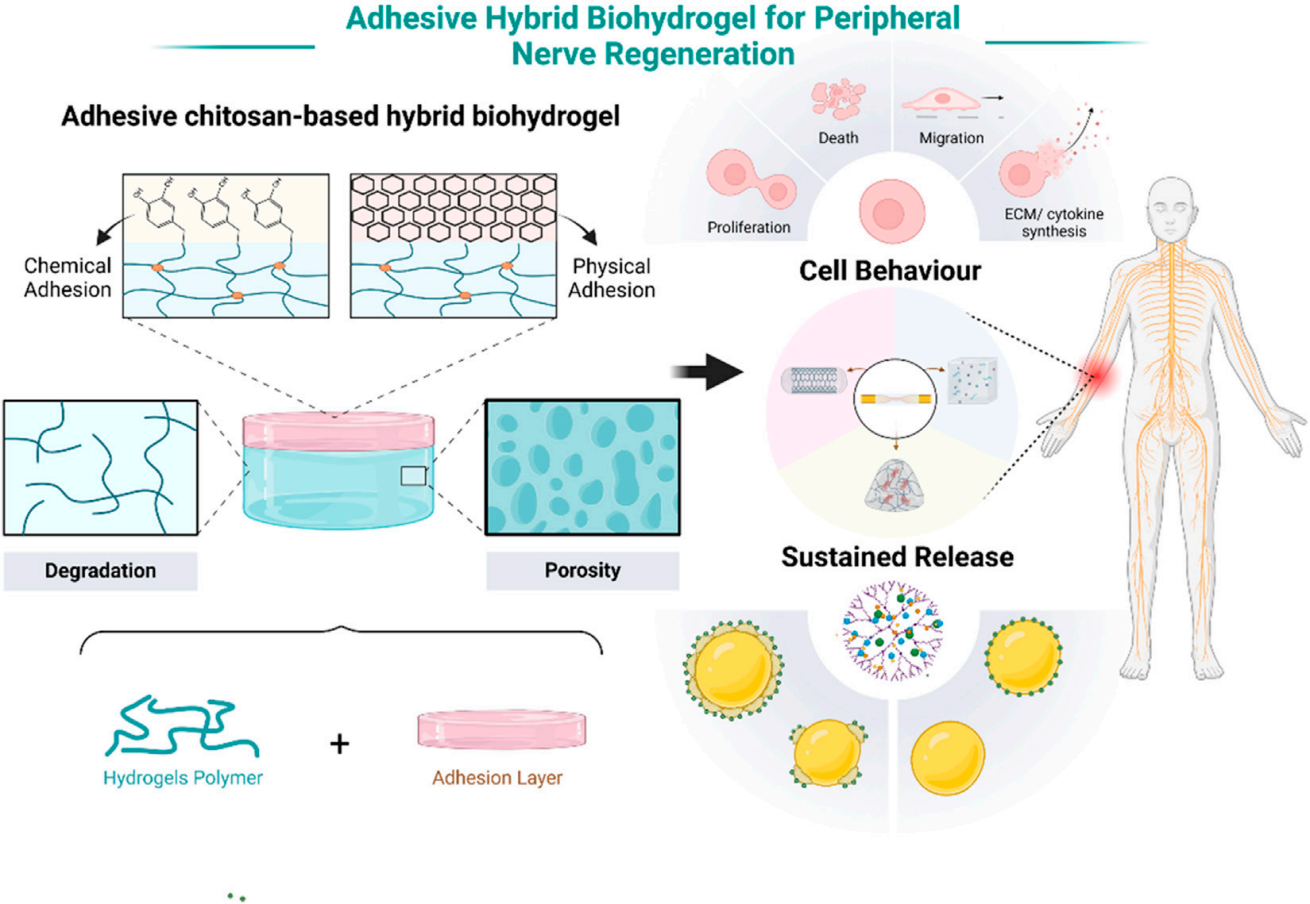
Schematic illustration of the adhesive chitosan-based hybrid biohydrogels for peripheral nerve injury repair. Note: Created in BioRender.com.

Adhesive chitosan-based hybrid biohydrogels are currently a focal point in the treatment of peripheral nerve injuries due to their unique properties and therapeutic potential. These hydrogels are composed of chitosan, a biocompatible polymer, integrated with other biomaterials to form a supportive, adhesive matrix. The primary advantage of these hydrogels lies in their ability to promote neuronal cell regeneration and provide sustained release of growth factors or pharmaceuticals directly at the injury site (Figure 1). This dual functionality not only accelerates nerve repair but also reduces the need for microsurgical suturing, thereby simplifying the surgical process. The adhesive nature of these hydrogels ensures close contact with nerve tissues, facilitating better integration and reducing the mechanical stress typically associated with conventional nerve repair techniques. This innovative approach is transforming the way peripheral nerve injuries are treated, offering a less invasive and more efficacious alternative to traditional methods.

## 2 Chitosan-based hybrid biohydrogels

Chitosan-based hybrid biohydrogels have demonstrated multiple innovative designs in the field of tissue engineering and regenerative medicine, making them a current research focus. First, by incorporating conductive materials such as graphene, carbon nanotubes, or conductive polymers with chitosan, conductive hydrogels are formed, which can effectively promote tissue repair under electrical stimulation, particularly showing remarkable results in nerve and cardiac tissue regeneration (Amalakanti et al., 2024; Fang et al., 2024; Teng et al., 2023). Moreover, chitosan hydrogels can load growth factors, such as basic fibroblast growth factor (bFGF) or vascular endothelial growth factor (VEGF), to regulate cell proliferation and differentiation, thereby enhancing tissue regeneration (Alizadeh et al., 2019; Boido et al., 2019; Liu et al, 2022; Rao et al., 2020). At the same time, chitosan-based hydrogels are capable of loading stem cells, such as mesenchymal stem cells (MSCs) or neural stem cells (NSCs), providing a three-dimensional scaffold to maintain cell viability and further improve tissue repair outcomes (Bagher et al., 2019; Liu et al, 2020; Mahya et al., 2021; Salehi et al., 2019). These innovative designs not only enhance the adaptability of chitosan hydrogels in complex biological environments but also significantly expand their potential applications in various tissue repair fields, demonstrating promising prospects for the future.

### 2.1 Conductive hybrid biohydrogel

**FIGURE 2:**
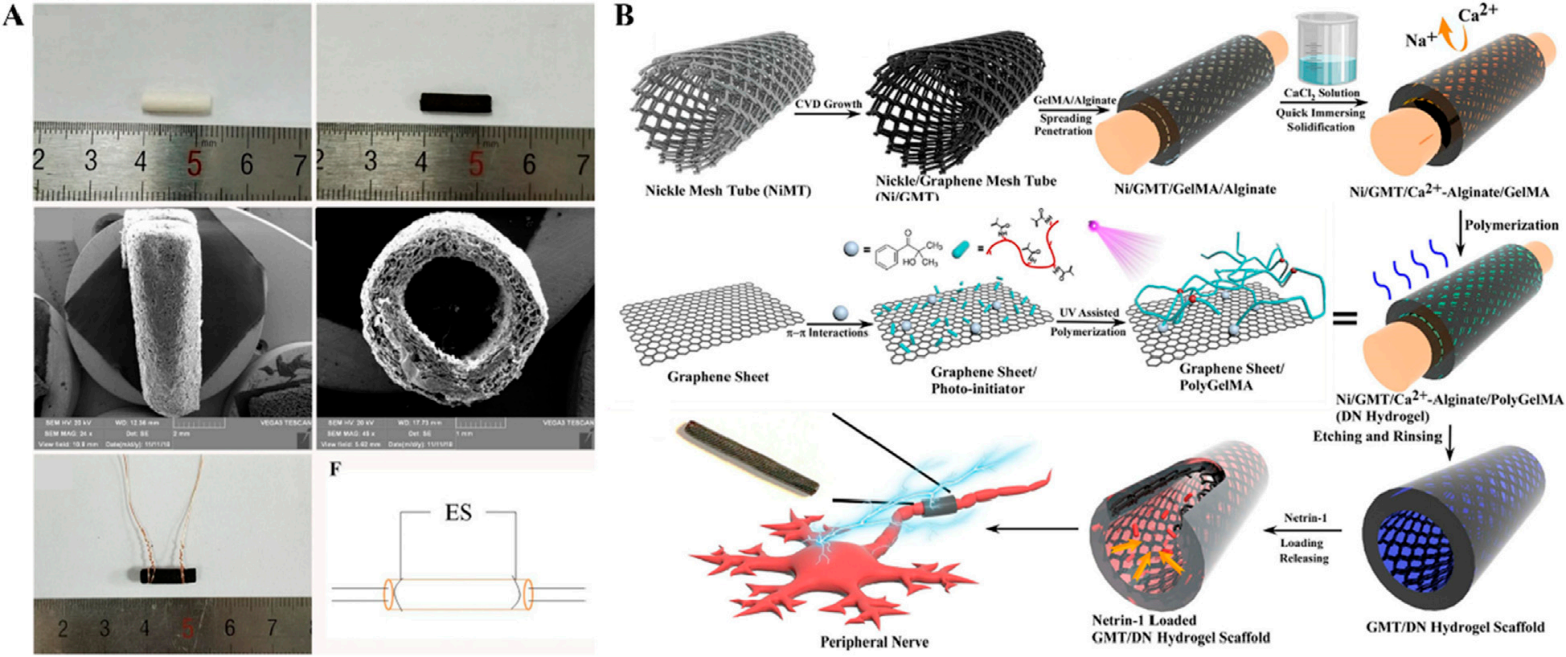
Studies on conductive hybrid biohydrogels **(A)** General observation images of HSPS-ES conduit with electrodes (Figures reproduced from Wu et al, 2020). **(B)** Schematic of netrin-1-loaded GMT/hydrogel conduit preparation (Figures reproduced from Huang et al., 2021). Reprinted (adapted) with permission from {Applied Materials}. Copyright {2021} American Chemical Society.

Conductive hybrid biohydrogels offer a significant advantage in peripheral nerve injury repair due to their ability to facilitate bioelectrical signal transmission, a crucial factor in nerve regeneration. As shown in Figure 2, The incorporation of conductive materials, such as polypyrrole, graphene, or carbon nanotubes, allows these hydrogels to replicate the native electrical conductivity of nerve tissues, which is essential for maintaining neuronal communication and promoting axonal growth (Lee et al, 2022; Lu et al., 2014; Ng et al., 2020). This electrical conductivity enhances cell signaling pathways that regulate Schwann cell activity, myelination, and synaptic plasticity, all of which are critical for functional nerve recovery (Runge et al., 2010; Yang et al., 2019). Moreover, conductive hydrogels can provide electrical stimulation to the injury site, further boosting cell proliferation and accelerating tissue regeneration (Bi et al., 2024; Huang et al., 2021; Wu et al, 2020). Compared to non-conductive hydrogels, the conductive variants significantly improve both the speed and quality of nerve repair, offering a more effective solution for clinical applications. This innovative design approach not only promotes biological integration but also supports the long-term functionality of the regenerated nerve, addressing key limitations of current repair strategies.

### 2.2 Hybrid biohydrogel with growth factors

**FIGURE 3:**
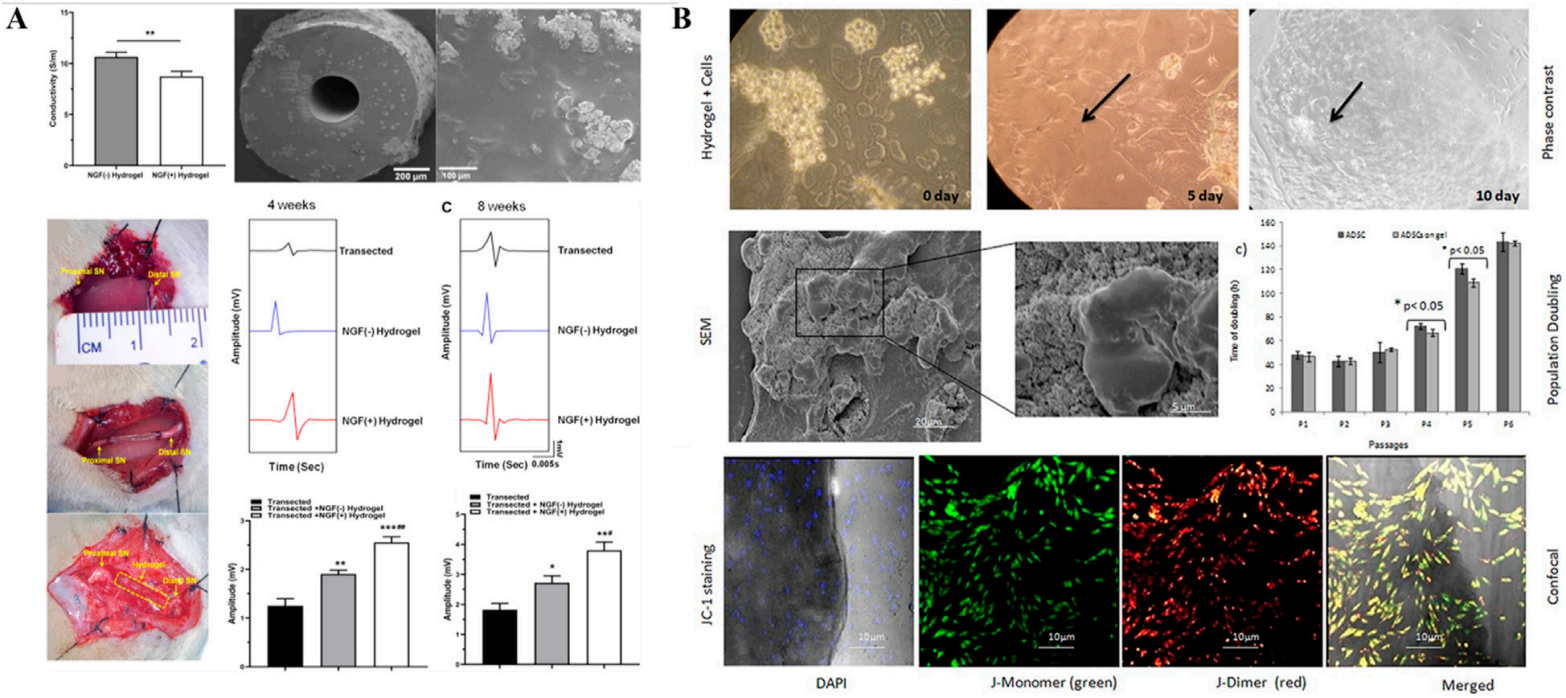
**(A)** Hydrogel tissue-engineered nerve conduits loaded with growth factors. (Figures reproduced from Su et al., 2023). Reprinted (adapted) with permission from {ACS Applied Bio Materials}. Copyright {2023} American Chemical Society. **(B)** Hydrogel tissue-engineered nerve conduits loaded with stem cells. hADSCs cultured on chitosan hydrogel on different days, the arrows in bright field image shows actively growing stem cells present in the hydrogel (Figures reproduced from Debnath et al., 2015).

Hybrid hydrogels loaded with growth factors have shown great potential in repairing peripheral nerve injuries (PNI) due to their ability to provide a bioactive scaffold that mimics the natural extracellular matrix. Growth factors such as nerve growth factor (NGF), brain-derived neurotrophic factor (BDNF), and glial cell-derived neurotrophic factor (GDNF) are essential in promoting neural survival, axonal growth, and remyelination (Kostiuchenko et al., 2024; Yan et al., 2012). Loading these factors into hydrogels enables their controlled and sustained release, creating an optimal environment for nerve regeneration over extended periods. This design feature significantly enhances therapeutic outcomes by preventing the rapid degradation of growth factors and ensuring their availability at the injury site (Kim et al., 2013). Furthermore, the hybrid nature of the hydrogels, combining both natural and synthetic polymers, provides enhanced mechanical strength, biocompatibility, and degradation rates, which are critical for adapting to the dynamic environment of peripheral nerve tissues (Choi et al., 2018) (Figure 3A). The combination of these properties allows for the functional restoration of damaged nerves, making growth factor-loaded hybrid hydrogels a highly effective platform for PNI repair.

### 2.3 Hybrid biohydrogel with stem cells

Hybrid hydrogels loaded with stem cells have emerged as an innovative solution for peripheral nerve injury (PNI) repair due to their ability to deliver regenerative cells in a controlled and supportive environment. Stem cells, such as mesenchymal stem cells (MSCs) or neural stem cells (NSCs), are known for their capacity to differentiate into various cell types, including neurons and Schwann cells, which are essential for nerve regeneration (Huang et al., 2017). As illustrated in Figure 3B, by embedding stem cells within hydrogels, researchers can ensure localized delivery and protection of these cells, preventing their rapid degradation or immune rejection (Debnath et al., 2015). This controlled release system enhances cell survival, proliferation, and differentiation at the injury site, leading to improved nerve repair outcomes. Hybrid hydrogels, which combine natural and synthetic polymers, offer advantages in terms of mechanical stability, biocompatibility, and tailored degradation rates (Li et al., 2014). This allows the hydrogel to gradually degrade as the nerve tissue regenerates, reducing the need for surgical removal (Frazier et al., 2021). The combination of stem cell delivery and the supportive scaffold properties of the hydrogel makes this approach a highly effective strategy for enhancing functional nerve recovery in PNI.

## 3 Adhesive between hydrogel and epineurial tissue

The epineurium covers the surface of nerve axons, and the adhesive properties of the interface between the hydrogel and the epineurium directly influence the effectiveness and biocompatibility of its application in the aforementioned areas. Since the 1980s, epineural adhesives have been developed to adhere to nerve surfaces for drug delivery. Common epineural adhesives include polyacrylates, alginates, chitosan, and cellulose derivatives. They are processed into dry forms (tablets, films, and patches) and wet forms (hydrogels and ointments). When hydrogel adhesives come into contact with the epineurium, the polymer chains within the hydrogel interpenetrate with the glycoproteins in the mucosa, allowing the hydrogel adhesive to tightly adhere to the epineurium. Therefore, robust interfacial adhesion between the hydrogel and epineural tissue is one of the key factors ensuring the overall stability and reliability of the hydrogel during its application.

### 3.1 Chemical structure adhesion interface

Biohydrogels can be bridged to the epineurium through the inclusion of specific chemical structures, such as catechol or other substances that contain catechol groups, such as epigallocatechin gallate (EGCG) and tannic acid (TA).

**FIGURE 4:**
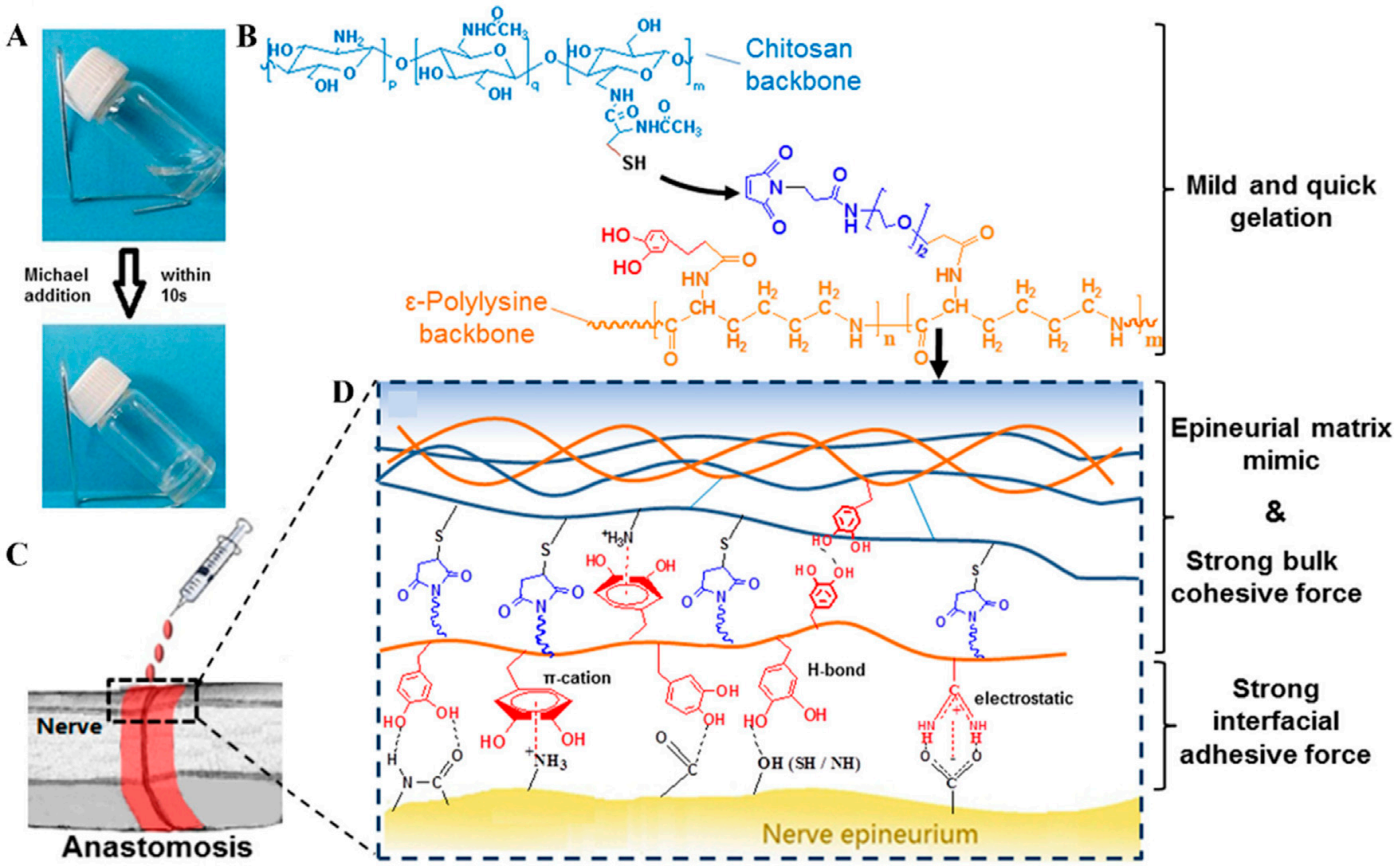
Biohydrogel adhesion based on catechols groups. **(A)** Formation of Hydrogel Network; **(B)** Gelation Mechanism through Michael Addition; **(C)** Schematic of Nerve Anastomosis Test; **(D)** Schematic of Interactions inside the Adhesives (Figures reproduced from Zhou et al., 2016).

In 1981, Waite and Tanzer first discovered a catechol-based adhesion mechanism in the adhesive feet of mussels (Waite and Tanzer, 1981). Subsequently, many adhesives containing phenolic components were reported (Figure 4) (Yang et al., 2021; Zhou et al., 2021), but most of these adhesives could only adhere in dry conditions and were not suitable for moist environments filled with tissue fluids found inside the body (Hu et al., 2018; Priemel et al., 2021). Catechol plays a crucial role in adhesion, and the synergistic action between various amino acids and catechol in the adhesive proteins of mussels and sandcastle worms makes underwater adhesion possible. For example, during the secretion process of the sandcastle worm’s glue, the pH shifts from weakly acidic to weakly alkaline, prompting the gradual oxidation of dopamine (DOPA) to DOPA-quinone, which accelerates the cohesive process of the adhesive proteins. The metal ions in seawater form complexes with the adhesive proteins, speeding up the curing of the adhesive. The hydrophobicity of the insoluble complex aggregates aids in water displacement, increasing the contact between the adhesive proteins and the substrate. The final adhesion is achieved through the interactions between dopamine and the substrate.

In recent years, researchers have delved into the adhesion mechanisms of mussels in wet environments and the properties of their adhesive proteins. The foot proteins Mfp-5 and Mfp-3 secreted by the mussel’s foot, located at the plaque surface, contain abundant DOPA and play a crucial role in underwater adhesion. The complexation and oxidation of catechol oxidase with DOPA, along with the marine weakly alkaline environment, increase the crosslinking density and cohesive strength of the adhesive. Unreacted dopamine adheres to substrates in water through various interactions. Additionally, the catechol groups undergo covalent auto-crosslinking under oxidizing conditions, significantly enhancing the cohesive strength of wet adhesives, while catechol groups help form a protein coagulation layer with excellent wettability on all substrates immersed in water. Recent studies have also found that catechol groups promote self-assembly through cation-π interactions and hydrogen bonding (Zhang et al, 2020), which facilitate instant adhesion and polymer coagulation.

### 3.2 Physical structure adhesion interface

In addition to adhesion based on catechol-related chemical bonds, mimicking mussels, and sandcastle worms, there are also examples of physical structure adhesion in nature, such as those found in geckos (Autumn et al., 2000; Tian et al., 2006), octopuses (Baik et al., 2017; Lee et al, 2022), tree frogs (Drotlef et al., 2013), and other insects (Liu et al, 2021). In recent years, the mechanism of adhesion formed by specially designed physical structures has received unprecedented attention, and many related studies have been published (Li et al., 2024). Adhesives can adhere to different surfaces through van der Waals forces, electrostatic forces, and hydrophobic/hydrophilic interactions.

Geckos can move freely on smooth surfaces because they provide strong and reversible adhesion on surfaces with varying roughness and orientation. This extraordinary adhesion ability is due to the millions of tiny setae (bristles) on gecko feet, which are divided into hundreds of even smaller nanoscale tips (spatulae). These tips form intimate contact with various surfaces through van der Waals forces, resulting in a strong adhesive force (approximately 10 N/cm^2^) (Jeong and Suh, 2009). Xue et al., inspired by gecko feet, designed and fabricated a polydimethylsiloxane (PDMS) micropillar array with radially oriented conical tips (PROST). By mimicking the unique movement pattern of gecko toes, PROST achieved excellent adhesion on both flat and spherical surfaces, with up to 150 adhesion cycles (Shi et al., 2022).

Octopuses can firmly adhere to the rocks on the ocean floor and move swiftly. Their outstanding adhesive ability primarily depends on their suckers, which enable their tentacles to attach to foreign surfaces and achieve flexible control and reversible movement with strong adhesive strength. Based on the adhesion mechanism of octopus suckers, Lee et al. designed a smart adhesive pad that can be actively controlled through a thermal-responsive drive, achieving reversible adhesion by adjusting the pressure difference inside and outside the pad cavity.

**FIGURE 5:**
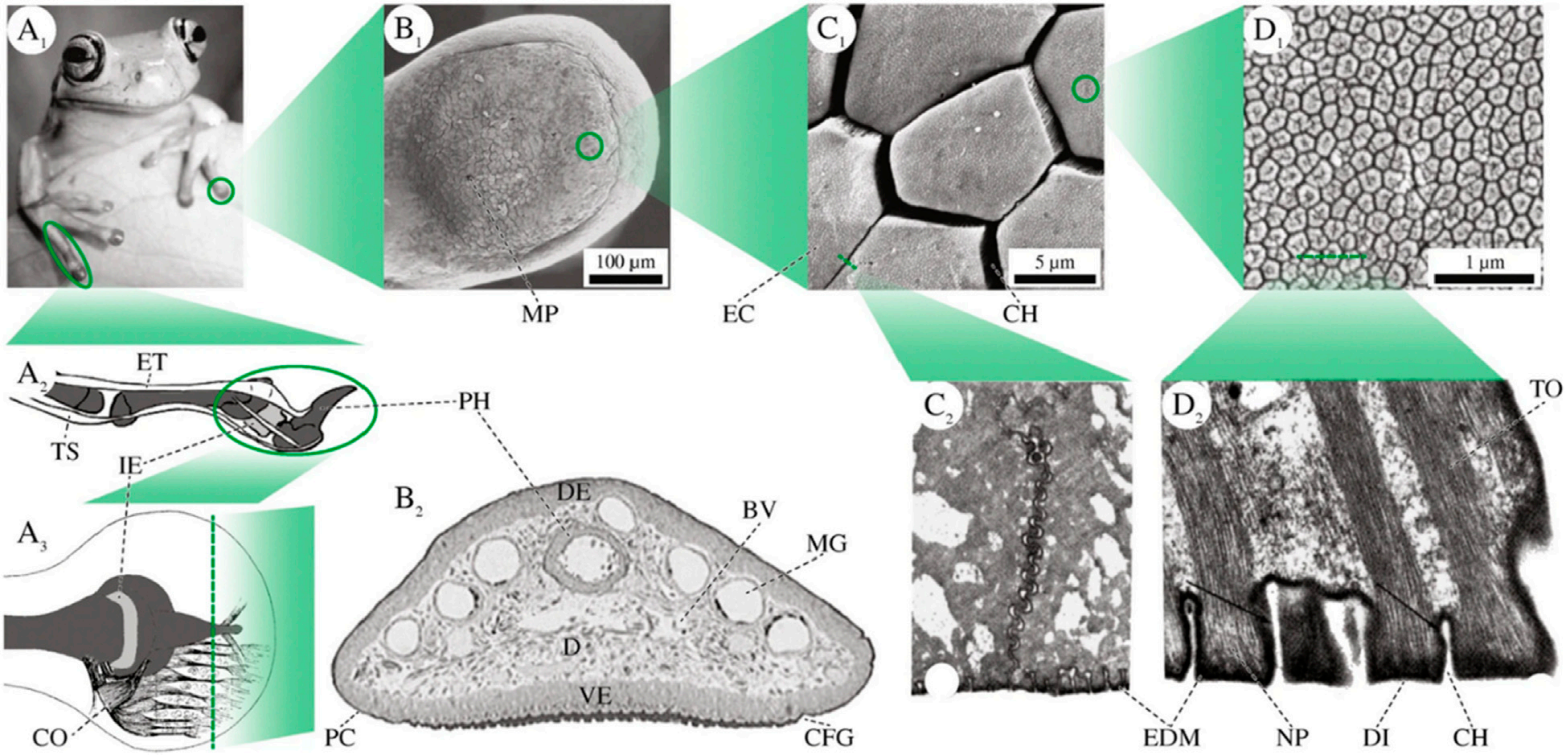
Physical structure adhesion in nature. **(A**_**1**_ **)** Forelimbs of Litoria caerulea. **(A**_**2**_ **)** Schematic lateral view of tendons, phalangi (dark grey), and the intercalary element (light grey) in a digit of Scinax squalirostris. **(A**_**3**_ **)** Schematic depiction of the collagen fibres in a pad of Hyla dominicensis in dorsal view. B Superficial and internal pad structures in L. caerulea. **(B**_**1**_ **)** SEM image of the ventral epidermis. **(B**_**2**_ **)** Transverse section through the toe of a juvenile frog. C Epidermal cells on the ventral surface. **(C**_**1**_ **)** SEM image of polygonal cells in L. caerulea. **(C**_**2**_ **)** TEM image of a tangential cross-section through the apical part of two adjacent cells in *Hyla cinerea*. D Fine structures of the apical surface of an epidermal cell. **(D**_**1**_ **)** High power SEM image of nanopillars and their central depressions (“dimples”) in L. caerulea. **(D**_**2**_ **)** TEM image of a cross-section through a row of nanopillars in *H. cinerea* (black arrows: EDM). BV blood vessels, CFG circumferential groove, CH channel between two epidermal cells/nanopillars, CO collagen fibres, D dermis, DE dorsal epidermis, DI dimple, EC epidermal cell, EDM electron dense material, ET extensor brevis profundus tendon, I.E., intercalary element, MG mucus gland, MP mucus pore, NP nanopillar, PC pad curvature, PH (terminal) phalanx, TO tonofilaments, TS tendo superficialis, VE ventral epidermis. The illustrations are not to scale (Figures reproduced from Langowski et al., 2018).

Tree frogs can firmly adhere to and successfully climb soft leaves, attributed to the specialized microstructures on the soles of their feet. These microstructures are composed of hexagonal epithelial cells, each side measuring 10 μm, separated by tiny channels (2 μm wide). These channels exhibit omnidirectional peel resistance as well as drainage on wet and rough surfaces, enabling direct contact for efficient adhesion (Figure 5) (Langowski et al., 2018; Liu et al, 2021). Xue et al., inspired by the structure of tree frog toes, designed a composite micropillar array with nanoscale pits on the surface. When pressure is applied, its adhesive force can reach 36.5 times that of tree frog toe pads (Liu et al, 2020).

The clingfish exhibits fast and reversible adhesion to various underwater substrates due to the presence of a suction disc with a hexagonal pattern separated by interconnected grooves, which enhances drainage capability. Inspired by the discontinuous hexagonal surface drainage structure of the clingfish, Long et al. combined an energy-dissipating hydrogel matrix with nanoscale dynamic bonds to propose a fast-adhesive, high-strength, and reversible wet adhesive (Rao et al., 2018).

Adhesion resulting from physical structures similar to those of tree frog toe pads, gecko feet, octopus suckers, and clingfish suction discs provides significant inspiration for the development of materials in fields such as biomedical engineering and wearable flexible electronics (Liimatainen et al., 2020; Zhang et al, 2020).

## 4 Advantages of adhesive chitosan-based hybrid biohydrogels

Adhesive hybrid biohydrogels present a promising avenue for peripheral nerve injury repair, harnessing several unique advantages. Firstly, these hydrogels offer tunable degradation rates through molecular chain composition adjustments, allowing for a tailored approach to nerve healing processes. Secondly, they facilitate the controlled release of therapeutic drugs and factors, ensuring sustained delivery at the injury site, which is crucial for effective regeneration. Thirdly, the incorporation of conductive materials into the hydrogels enables the fabrication of conductive sheaths that enhance neural regeneration by improving electrical signal transmission across the damaged area. Additionally, the adhesive nature of these hydrogels simplifies the application process, reducing the complexity and need for microsurgical suturing, thereby lessening the dependency on expensive microsurgical equipment. Lastly, by minimizing the need for extensive suturing, these hydrogels reduce the risk of further neural damage caused by the suturing process itself. Overall, adhesive hybrid biohydrogels represent a significant advancement in peripheral nerve repair, offering improved outcomes through innovative material science and engineering approaches.

### 4.1 Regulate the degradation rate

By modifying the ratio of natural and synthetic polymers, the mechanical properties and degradation speed of the hydrogels can be finely controlled, allowing for a customized response to the biological environment. This tunability is critical for nerve regeneration, as it ensures that the hydrogel provides structural support for the regenerating nerve over an appropriate time frame and degrades as the nerve tissue heals. Studies have demonstrated that these materials can be optimized to match the degradation rate to the nerve healing process, thereby promoting more effective regeneration compared to traditional materials (Lan et al., 2023; Zhao et al., 2017). The ability to control degradation kinetics by manipulating molecular structure offers a significant advantage in the field of nerve repair.

### 4.2 Sustained release drugs or factors

Adhesive hybrid biohydrogels offer a unique advantage in regulating the controlled release of therapeutic drugs or factors, making them particularly effective in the repair of peripheral nerve injuries. These hydrogels combine both natural and synthetic materials to create a biocompatible environment that adheres well to damaged tissues, improving drug retention at the injury site. The adhesive properties of these materials not only promote cell attachment and tissue integration but also enable precise control over the release rates of bioactive molecules. This controlled release is critical in nerve regeneration, as it ensures the sustained presence of neurotrophic factors or anti-inflammatory agents that can enhance neural repair. Studies have shown that tuning the mechanical and chemical properties of hybrid biohydrogels can influence the degradation rate and, consequently, the drug release profile (Gnavi et al., 2017; Li et al., 2018; Ning et al., 2019). This capability allows for customization depending on the type of injury and desired therapeutic outcome (Liu et al, 2021; Wang et al, 2023; Xu et al., 2022). Furthermore, the application of these biohydrogels in nerve repair demonstrates enhanced nerve growth and functional recovery due to their biomimetic properties (Wang et al, 2023). Their versatility in delivering both small molecules and larger proteins further supports their use in complex nerve injury scenarios (Kulkarni et al., 2021). In conclusion, adhesive hybrid biohydrogels represent a promising platform for nerve regeneration due to their ability to modulate drug release and promote tissue healing.

### 4.3 Conductive hybrid biohydrogel

These hydrogels combine both biocompatible polymers and conductive elements, such as graphene or polypyrrole, which mimic the natural electrical environment of nerve tissues. This electrical conductivity is critical for promoting the growth and alignment of nerve cells, which are essential for functional recovery in nerve injuries. Conductive biohydrogels not only provide a scaffold for cell attachment and migration but also enable electrical stimulation, which has been shown to accelerate neural differentiation and neurite outgrowth (Cheng et al., 2024). The incorporation of conductive materials further enhances the bioadhesive properties of these hydrogels, ensuring close contact with injured nerve tissues and improving the precision of therapeutic delivery (Aruã Clayton Da et al., 2023; He et al., 2020; Xu et al., 2023; Zhang et al., 2022). Studies have demonstrated that conductive hybrid hydrogels can enhance the regeneration of peripheral nerves by promoting electrical signaling between damaged neurons and supporting cells (Burdick et al., 2006; Lu et al., 2018; Park et al., 2010; Ramos Ferrer et al., 2024). Additionally, these materials improve the delivery of neurotrophic factors, which further accelerates the regeneration process (Peressotti et al., 2021; Su et al., 2023; Xu et al., 2024). Conductive hydrogels' versatility and multifunctionality make them highly suitable for the complex environment of nerve repair (Deng et al., 2022; Liu et al., 2017; Xuan et al., 2023). In conclusion, conductive adhesive hybrid biohydrogels present a promising solution for enhancing peripheral nerve regeneration by combining structural support with electrical conductivity.

### 4.4 Reduce the difficulty of surgery

Adhesive hybrid biohydrogels are emerging as a transformative solution in peripheral nerve repair, primarily due to their ability to bridge repair materials and the epineurium through adhesive interfaces. This novel approach significantly simplifies surgical procedures by reducing the reliance on intricate microsurgical techniques and costly suturing instruments. The integration of bioadhesive properties in hydrogels facilitates a seamless interface with biological tissues, enhancing the stability and integration of the repair site. Such hydrogels are designed to mimic the mechanical and biochemical cues of the nerve environment, which are crucial for promoting axonal growth and nerve regeneration (Fan et al., 2022; Feng et al., 2016; Xiao et al., 2023). Studies by Zhang et al. highlight the enhanced regenerative outcomes associated with these bioadhesives, as they provide both structural support and biochemical signaling necessary for effective nerve healing (Zhang et al., 2023). Further, Zhou et al. demonstrate the reduced surgical time and improved handling characteristics compared to traditional methods (Zhou et al., 2023). Research by Liu and Wu emphasizes the reduced immunogenicity and enhanced biocompatibility of these materials, which are essential for long-term success in nerve repair (Liu et al., 2012; Wu et al, 2020). Lastly, a comprehensive review by Asser et al. provides a meta-analysis of clinical trials, showcasing significant advancements in patient outcomes due to the adoption of adhesive hybrid biohydrogels in peripheral nerve surgeries (Sallam et al., 2022). These developments mark a pivotal shift towards less invasive and more efficient neurosurgical procedures, promising improved recovery rates and functionality in nerve repair.

### 4.5 Avoid suture-related damage

These innovative hydrogels minimize the mechanical trauma to nerves, which is often inevitable with traditional suturing techniques. By providing a seamless and gentle integration with nerve tissues (Spearman et al., 2018). Studies such as those by Lin et al. and Jha et al. have documented a significant reduction in the expression of pro-inflammatory cytokines, such as TNF-α and IL-6, in nerve repair scenarios utilizing these biohydrogels (Jha et al., 2023; Lin et al., 2009). This decrease in inflammatory mediators not only protects the nerve from secondary damage but also creates a conducive environment for nerve fiber regeneration. Furthermore, research by Ramos et al. has shown that these hydrogels can release neurotrophic factors that actively promote axonal growth and myelin sheath formation (Ramos Ferrer et al., 2024). A study by DiStefano and Iatridis highlights the role of these hydrogels in reducing fibrosis around the repair site, a common complication that can impair nerve function (DiStefano et al., 2020). Finally, a comprehensive review by Fedak et al. synthesizes findings from multiple clinical trials, confirming the enhanced functional recovery in patients treated with adhesive hybrid biohydrogels compared to those undergoing conventional microsurgical techniques (Fedak et al., 2011). The adoption of these bioadhesive materials marks a significant advance towards less invasive and more effective therapies for nerve injuries.

## 5 Summary and prospect

Adhesive chitosan-based hybrid biohydrogels exhibit significant advantages and promising prospects in the repair of peripheral nerve injuries. Firstly, these hydrogels provide an ideal scaffold and microenvironment for nerve regeneration. Their biocompatibility and three-dimensional structural properties support the adhesion and growth of nerve cells, promoting the recovery of damaged nerves. Additionally, chitosan-based materials effectively promote the proliferation, migration, and reprogramming of nerve cells, which are essential for accelerating nerve repair. Moreover, chitosan-based hydrogels can act as drug delivery carriers, allowing the sustained release of neurotrophic factors and other nerve growth drugs. This prolonged retention at the site of injury enhances drug bioavailability and significantly promotes nerve regeneration. The natural antibacterial properties of chitosan add another layer of advantage to the application of hydrogels. By preventing post-implantation infections and reducing inflammatory responses, chitosan-based hydrogels create a more favorable environment for nerve regeneration. These characteristics make them a multifunctional repair material, not only speeding up nerve repair but also reducing the incidence of postoperative complications.

The adhesive properties of chitosan-based hybrid biohydrogels are a major highlight in nerve repair. Through carefully designed adhesive interfaces, these hydrogels can seamlessly bridge with the epineurium, avoiding the physical damage to nerves caused by traditional suturing methods. Adhesion can be achieved through chemical modifications or physical structural designs, such as surface modification or the introduction of specific functional groups to enhance the interaction between the hydrogel and nerve tissue. This non-invasive adhesive repair method reduces inflammation and minimizes additional damage to nerve tissues, further improving the efficiency of nerve repair.

Overall, adhesive chitosan-based hybrid biohydrogels provide an innovative and highly promising material design strategy for peripheral nerve regeneration. Their multifunctionality—from providing structural support to promoting cell growth, to drug delivery and antibacterial action—offers new insights and directions for future nerve regeneration research and clinical applications. As research into the properties of chitosan-based materials deepens, their application in peripheral nerve injury repair is expected to expand, potentially leading to significant advancements in regenerative medicine.
